# Supplementary material for: The hagfish genome and the evolution of vertebrates
Source: Nature. 2024 Jan 23;627(8005):811–20. doi: 10.1038/s41586-024-07070-3 (PMC10972751; doi:10.1038/s41586-024-07070-3)

nature portfolio

https://doi.org/10.1038/s41586-024-07070-3

Supplementary information

The hagfish genome and the evolution of vertebrates

In the format provided by the authors and unedited

Supplementary Note 1. Phylogenomics and support of cyclostomes

While many shared characteristics between lamprey and hagfish prompted to associate them in a cyclostome clade[1], more rigorous cladistic morphological analyses incorporating fossil evidence later questioned the validity of this clade[2]. Cyclostome monophyly has been supported by 18S and 28S since their use in phylogenetic analysis became prominent in the 1990s'[3–5]. Analysis of mitochondrial DNA also supported the same topology[6,7]. Later, the availability of transcriptomic data through EST sequencing or RNA-seq has promoted multigene phylogenies, which provides enhanced phylogenetic signal and limits long-branch attraction problems. Phylogenomic studies also confirmed the monophyly of cyclostomes[8–12]. The examination of microRNA complement also provided support for the monophyly of cyclostomes[13], but the amenability of this character was later questioned because of the possibility of homoplasy[14]. Recently, discovery of a new hagfish fossil refined the interpretation of hagfish characters and provided morphological evidence for the monophyly of cyclostomes[15]. However, these studies did not account for some potential issues affecting phylogenetic reconstruction:

I. The occurrence of multiple rounds of whole genome duplication in cyclostomes, of which two are independent from gnathostomes, could make some of the single-copy orthologous defined using gnathostome genomes prone to paralogue issues, such as persistence of duplicates, or hidden paralogy with independent losses[16]. Even if such problems are difficult to deal with, we suggest that performing a *de novo* orthology reconstruction applying an established and robust method (OMA) to a set of proteome including two lamprey and one hagfish could help limit such problems[17].

II. Lampreys and hagfish show an increased evolutionary rate compared to other vertebrates, and the closest vertebrate outgroup, tunicates is also very fast evolving, which altogether creates a context prone to long-branch attraction. To tackle long-branch attraction, enhanced taxonomic sampling is essential, but also applying the most parameter rich site-heterogenous models. Notably, most existing phylogenetic analyses were performed with only one representative of lamprey (*Petromyzon marinus*) and one of hagfish (*Eptatretus burgeri*) and only employed CAT+Poisson model and not CAT+GTR which is more computational intensive but also provided better results in resolving animal phylogeny[18].

III. Recently, it was also demonstrated that composition differences between lineages can have an adverse effect on phylogenetic reconstruction, even when amino-acid are employed[19]. A possible solution is amino-acid recoding in broader functional categories (e.g. Dayhoff 6 categories). As lamprey (and to a lesser extent hagfish) were known to be GC-rich genomes, we appreciated that composition could be an important confounding factor in promoting cyclostome monophyly that deserved to be examined[11,20].

In order to address these issues in the light of the hagfish genome, we constructed a set of 1447 orthologous genes (~eight-fold more than previously analysed) by applying OMA to selected genomes. These phylogenetic markers were then extracted from a set of transcriptomes gathering the largest taxonomic sampling ever considered for both hagfishes and lampreys. Notably, we included both representatives of the Pacific (genus *Eptatretus*) and Atlantic (genus *Myxine*) hagfishes, as well as for lampreys, representative of the genus *Mordacia* and *Geotria*, which are early diverging lamprey lineages. Cyclostome monophyly was recovered, first with a partitioned analysis of all genes

using maximum likelihood (**Extended Data Fig. 2a**), then by selecting a subset of orthologous genes (176) showing the lowest saturation levels, and applying the site-heterogeneous model CAT+GTR (**Extended Data Fig. 2b**). Finally, we employed six-category amino-acid recoding to alleviate compositional bias, which still supports cyclostome monophyly (**Extended Data Fig. 2c**). Posterior predictive tests (**Extended Data Fig. 2d**) indicate that recoding strongly enhances model adequacy regarding compositional heterogeneity.

Supplementary Note 2: 18 ancestral chordate/proto-vertebrate linkage groups

Historically there has been persistent confusion around the reconstruction of ancestral ("proto") vertebrate chromosomes based on comparative genomics[21–27]. This has been resolved with the advent of chromosome-scale genome sequences for amphioxus, lamprey, and diverse invertebrates leading to a consensus[25,26] of 18 ancestral chordate or proto-vertebrate linkage groups. (These are often colloquially referred to as "ancestral chromosomes" but may represent ancestral chromosome arms.)

Here we use the 18 ancestral stem-vertebrate linkage groups previously defined by ref. [26] based on a comprehensive analysis of bilaterian chromosome evolution, including multiple outgroups. These units were designated as A1, A2, and B-Q, which is the terminology that we adopt here. The use of multiple outgroups in this analysis makes this a definitive and stable reconstruction of these stem-vertebrate linkage groups.

These 18 linkage groups are in one-to-one correspondence with the 18 "proto-vertebrate chromosomes" ("Pvcs") of ref. [25]. In particular CLGs B-Q the same as Pvcs 1-16 of ref. [25] (as noted in the Supplementary Note 3.1.2 and Supplementary Table 9 of [25]). These groups were previously described in ref. [24], and are in 1:1 correspondence with early analyses based on sub-chromosomal assemblies of amphioxus[27].

The remaining two stem-vertebrate linkage groups A1 and A2 of ref. [26] were derived from CLGA of ref. [24] based on the finding that these two groups are consistently (and therefore ancestrally) separate in multiple other bilaterians but are fused-and-mixed in amphioxus. The distinctness of these two groups on the vertebrate stem was first recognized by ref. [25] based on comparative analysis including lamprey genomes. A1 and A2 of ref. [24] correspond to Pvc17 and Pvc18 of ref. [25], respectively. This is evident, for example, from comparisons to scallop chromosomes used in these two studies. (Please note that in Figure 2 of ref. [25], 18 groupings of amphioxus (sub-chromosomal) scaffolds are shown, following a clustering approach similar to ref. [27]. These groupings are not chromosomes, and relationship between these clusters of scaffolds and the 19 chromosomes of amphioxus is discussed in ref. [24]. Similar considerations hold for the clustering of *Trichoplax* scaffolds shown in that figure, which correspond to scaffold clusters following ref. [28] and do not represent chromosomes.)

Finally, the relatively small ancestral linkage group A2 (or Pvc18) has several anomalous properties, as noted in ref. [25]. It is (1) poorly represented in gnathostomes (2) could not be segmented by their method due to extensive fragmentation in lampreys, and (3) was excluded from their analysis of lamprey hexaploidy. The absence of consistently linked genes from A2 in gnathostomes prevented us from including this linkage group in our molecular phylogenetic analysis of paralogons. Thus

although there are 18 ancestral stem-vertebrate linkage groups, we report only 17 paralogon trees (A1 and B-Q).

Supplementary Note 3: Identification, quantification and validation of germline-enriched repeats

Among vertebrates, hagfish were the first species recognized as undergoing programmed DNA elimination[29–35] and early studies identified a large number of satellite repetitive elements that are highly enriched within the germline-specific chromosomes/regions and represent a large fraction of eliminated chromatin across hagfish species[36–39]. While the process of somatic DNA elimination has been described in lamprey embryos[29–35], it has not been studied in hagfish due to the relative inaccessibility of early stage embryos. Analyses from lampreys suggest that programmed DNA elimination acts to repress the somatic expression of genes related to germline/pluripotency functions, and may partially resolve fundamental genetic conflicts between germline and soma [32–35].

To identify and quantify germline-enriched repeats, we compared DNA sequences from soma (blood) and germline (testes) that were sampled from the same individual to identify repetitive sequences and quantify their abundance in testes vs blood (**Methods**). In both hagfish and lamprey, the somatically eliminated chromosomes contain large numbers of highly repetitive satellite sequences[36–40] that are often not fully incorporated into genome assemblies and result in increased fragmentation of chromosomal assemblies for germline-specific regions[41]. We found that most germline-enriched repetitive elements belong to large repeat families consisting of highly similar (>%80 nucleic acid identity) sequences (**Fig. 5a,b, Extended Data Fig. 11m,n**). These include numerous novel repeat families as well as previously known germline-enriched satellite elements[36–39]. PCR validation confirms the presence and germline-specificity of these satellite elements (**Methods**). FISH hybridization to testes and blood interphase nuclei further demonstrates that these repetitive elements are highly enriched in germline, and are arrayed in dense tandem clusters that are often physically linked, and distributed across several distinct germline-specific regions (i.e., chromosomes) (**Fig. 5c, Extended Data Fig. 11**). Among our newly identified germline-specific repeats is the most abundant germline element yet to be identified in *E. atami* (HFR13), a 67 bp tandem repeat that spans >128 Mb, or ~3.7% of the germline genome (>9% of the germline-specific fraction). In total, the dozen highly abundant and validated repeats reported above span more than 30% of the germline-specific fraction.

References

1. Duméril, A. M. C. *Dissertation sur la famille des poissons cyclostomes, pour démontrer leurs rapports avec les animaux sans vertèbres*. (Didot, 1812).
2. Janvier, P. The phylogeny of the Craniata, with particular reference to the significance of fossil “agnathans.” *J. Vert. Paleontol.* **1**, 121–159 (1981).
3. Mallatt, J. & Sullivan, J. 28S and 18S rDNA sequences support the monophyly of lampreys and hagfishes. *Mol.*

Biol. Evol. **15**, 1706–1718 (1998).

4. Kuraku, S., Hoshiyama, D., Katoh, K., Suga, H. & Miyata, T. Monophyly of Lampreys and Hagfishes Supported by Nuclear DNA–Coded Genes. *J. Mol. Evol.* **49**, 729–735 (1999).
5. Stock, D. W. & Whitt, G. S. Evidence from 18S ribosomal RNA sequences that lampreys and hagfishes form a natural group. *Science* **257**, 787–789 (1992).
6. Delarbre, C., Gallut, C., Barriel, V., Janvier, P. & Gachelin, G. Complete Mitochondrial DNA of the Hagfish, Eptatretus burgeri: The Comparative Analysis of Mitochondrial DNA Sequences Strongly Supports the Cyclostome Monophyly. *Mol. Phylogenet. Evol.* **22**, 184–192 (2002).
7. Rasmussen, A. S., Janke, A. & Arnason, U. The mitochondrial DNA molecule of the hagfish (Myxine glutinosa) and vertebrate phylogeny. *J. Mol. Evol.* **46**, 382–388 (1998).
8. Takezaki, N., Figueroa, F., Zaleska-Rutczynska, Z. & Klein, J. Molecular phylogeny of early vertebrates: monophyly of the agnathans as revealed by sequences of 35 genes. *Mol. Biol. Evol.* **20**, 287–292 (2003).
9. Delsuc, F., Tsagkogeorga, G., Lartillot, N. & Philippe, H. Additional molecular support for the new chordate phylogeny. *Genesis* **46**, 592–604 (2008).
10. Delsuc, F., Brinkmann, H., Chourrout, D. & Philippe, H. Tunicates and not cephalochordates are the closest living relatives of vertebrates. *Nature* **439**, 965–968 (2006).
11. Kuraku, S. & Kuratani, S. Time scale for cyclostome evolution inferred with a phylogenetic diagnosis of hagfish and lamprey cDNA sequences. *Zoolog. Sci.* **23**, 1053–1064 (2006).
12. Blair, J. E. & Hedges, S. B. Molecular phylogeny and divergence times of deuterostome animals. *Mol. Biol. Evol.* **22**, 2275–2284 (2005).
13. Heimberg, A. M., Cowper-Sal-lari, R., Sémon, M., Donoghue, P. C. J. & Peterson, K. J. microRNAs reveal the interrelationships of hagfish, lampreys, and gnathostomes and the nature of the ancestral vertebrate. *Proc. Natl. Acad. Sci. U. S. A.* **107**, 19379–19383 (2010).
14. Thomson, R. C., Plachetzki, D. C., Mahler, D. L. & Moore, B. R. A critical appraisal of the use of microRNA data in phylogenetics. *Proceedings of the National Academy of Sciences* **111**, E3659–E3668 (2014).
15. Miyashita, T. *et al.* Hagfish from the Cretaceous Tethys Sea and a reconciliation of the morphological–molecular conflict in early vertebrate phylogeny. *Proc. Natl. Acad. Sci. U. S. A.* **116**, 2146–2151 (2019).
16. Kuraku, S., Meyer, A. & Kuratani, S. Timing of genome duplications relative to the origin of the vertebrates: did cyclostomes diverge before or after? *Mol. Biol. Evol.* **26**, 47–59 (2009).
17. Altenhoff, A. M. *et al.* OMA standalone: orthology inference among public and custom genomes and transcriptomes. *Genome Res.* **29**, 1152–1163 (2019).
18. Marlétaz, F., Peijnenburg, K. T. C. A., Goto, T., Satoh, N. & Rokhsar, D. S. A New Spiralian Phylogeny Places the Enigmatic Arrow Worms among Gnathiferans. *Curr. Biol.* **29**, 312-318.e3 (2019).
19. Feuda, R. *et al.* Improved Modeling of Compositional Heterogeneity Supports Sponges as Sister to All Other Animals. *Curr. Biol.* **27**, 3864-3870.e4 (2017).
20. Smith, J. J. *et al.* Sequencing of the sea lamprey (Petromyzon marinus) genome provides insights into vertebrate evolution. *Nat. Genet.* **45**, 415–21, 421e1-2 (2013).
21. Nakatani, Y., Takeda, H., Kohara, Y. & Morishita, S. Reconstruction of the vertebrate ancestral genome reveals dynamic genome reorganization in early vertebrates. *Genome Res.* **17**, 1254–1265 (2007).
22. Smith, J. J. & Keinath, M. C. The sea lamprey meiotic map improves resolution of ancient vertebrate genome duplications. *Genome Res.* **25**, 1081–1090 (2015).
23. Sacerdot, C., Louis, A., Bon, C., Berthelot, C. & Roest Crollius, H. Chromosome evolution at the origin of the ancestral vertebrate genome. *Genome Biol.* **19**, 166 (2018).
24. Simakov, O. *et al.* Deeply conserved synteny resolves early events in vertebrate evolution. *Nat Ecol Evol* **4**, 820–830 (2020).
25. Nakatani, Y. *et al.* Reconstruction of proto-vertebrate, proto-cyclostome and proto-gnathostome genomes provides new insights into early vertebrate evolution. *Nat. Commun.* **12**, 4489 (2021).
26. Simakov, O. *et al.* Deeply conserved synteny and the evolution of metazoan chromosomes. *Sci Adv* **8**, eabi5884 (2022).
27. Putnam, N. H. *et al.* The amphioxus genome and the evolution of the chordate karyotype. *Nature* **453**, 1064–1071 (2008).
28. Srivastava, M. *et al.* The Trichoplax genome and the nature of placozoans. *Nature* **454**, 955–960 (2008).

29. Kohno, S., Nakai, Y., Satoh, S., Yoshida, M. & Kobayashi, H. Chromosome elimination in the Japanese hagfish, Eptatretus burgeri (Agnatha, Cyclostomata). *Cytogenet. Cell Genet.* **41**, 209–214 (1986).
30. Nakai, Y. & Kohno, S. Elimination of the largest chromosome pair during differentiation into somatic cells in the Japanese hagfish, Myxine garmani (Cyclostomata, Agnatha). *Cytogenet. Genome Res.* **45**, 80–83 (1987).
31. Kohno, S.-I., Kubota, S. & Nakai, Y. Chromatin Diminution and Chromosome Elimination in Hagfishes. in *The Biology of Hagfishes* (eds. Jørgensen, J. M., Lomholt, J. P., Weber, R. E. & Malte, H.) 81–100 (Springer Netherlands, 1998). doi:10.1007/978-94-011-5834-3_6.
32. Smith, J. J., Antonacci, F., Eichler, E. E. & Amemiya, C. T. Programmed loss of millions of base pairs from a vertebrate genome. *Proc. Natl. Acad. Sci. U. S. A.* **106**, 11212–11217 (2009).
33. Smith, J. J., Baker, C., Eichler, E. E. & Amemiya, C. T. Genetic consequences of programmed genome rearrangement. *Curr. Biol.* **22**, 1524–1529 (2012).
34. Smith, J. J. *et al.* The sea lamprey germline genome provides insights into programmed genome rearrangement and vertebrate evolution. *Nat. Genet.* **50**, 270–277 (2018).
35. Bryant, S. A., Herdy, J. R., Amemiya, C. T. & Smith, J. J. Characterization of Somatically-Eliminated Genes During Development of the Sea Lamprey (Petromyzon marinus). *Mol. Biol. Evol.* **33**, 2337–2344 (2016).
36. Kubota, S., Kuro-o, M., Mizuno, S. & Kohno, S. Germ line-restricted, highly repeated DNA sequences and their chromosomal localization in a Japanese hagfish (Eptatretus okinoseanus). *Chromosoma* **102**, 163–173 (1993).
37. Goto, Y., Kubota, S. & Kohno, S. Highly repetitive DNA sequences that are restricted to the germ line in the hagfish Eptatretus cirrhatus: a mosaic of eliminated elements. *Chromosoma* **107**, 17–32 (1998).
38. Nabeyama, M., Kubota, S. & Kohno, S.-I. Concerted Evolution of a Highly Repetitive DNA Family in Eptatretidae (Cyclostomata, Agnatha) Implies Specifically Differential Homogenization and Amplification Events in Their Germ Cells. *Journal of Molecular Evolution* vol. 50 154–169 Preprint at https://doi.org/10.1007/s002399910017 (2000).
39. Kojima, N. F. *et al.* Whole chromosome elimination and chromosome terminus elimination both contribute to somatic differentiation in Taiwanese hagfish Paramyxine sheni. *Chromosome Res.* **18**, 383–400 (2010).
40. Timoshevskiy, V. A., Timoshevskaya, N. Y. & Smith, J. J. Germline-Specific Repetitive Elements in Programmatically Eliminated Chromosomes of the Sea Lamprey (Petromyzon marinus). *Genes* **10**, (2019).
41. Timoshevskaya, N. *et al.* An improved germline genome assembly for the sea lamprey Petromyzon marinus illuminates the evolution of germline-specific chromosomes. *Cell Rep.* **42**, 112263 (2023).

CLGA1

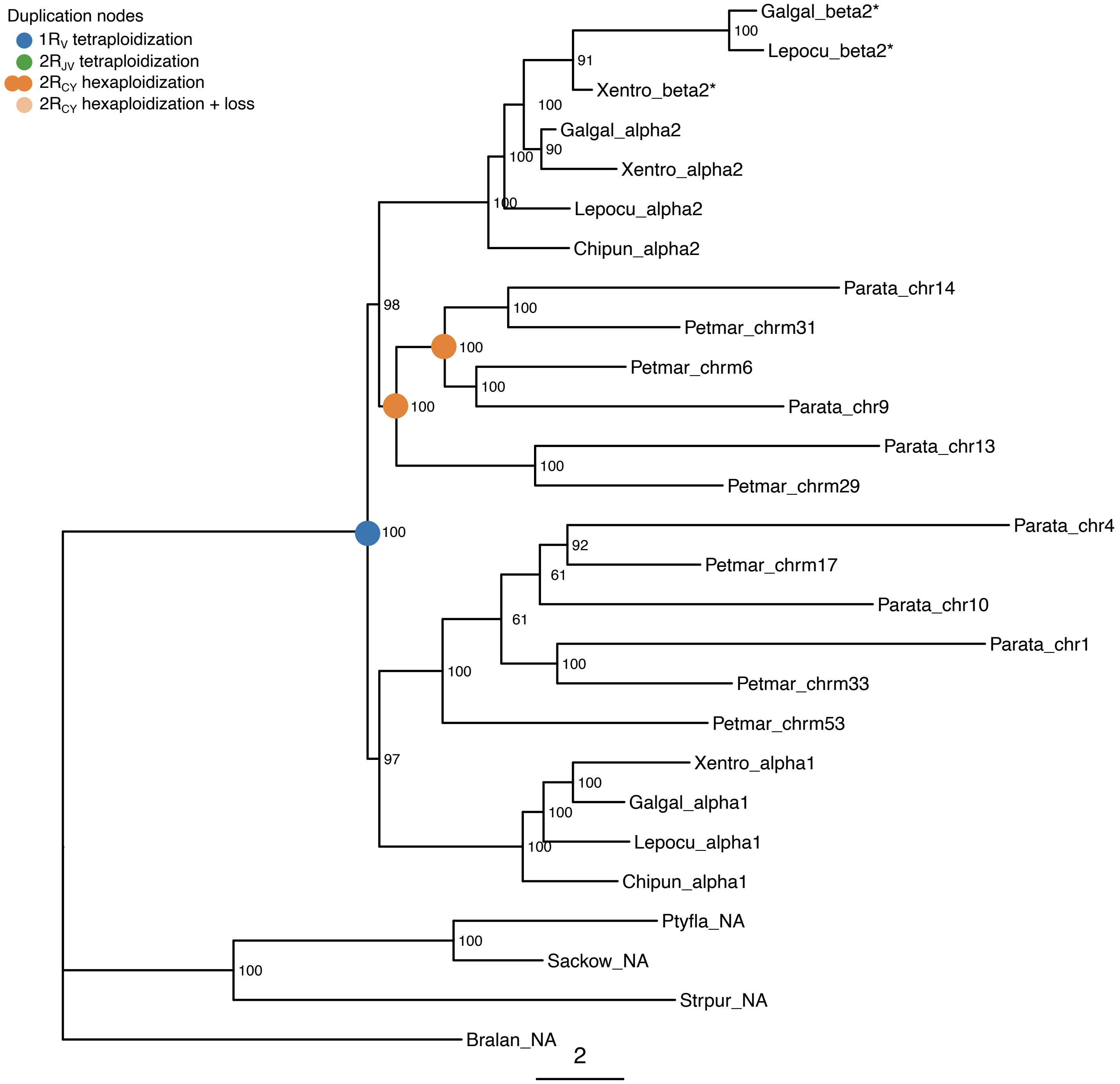
Duplication nodes
1RV tetraploidization
2RJV tetraploidization
2RCY hexaploidization
2RCY hexaploidization + loss
Galgal_beta2*
Lepocu_beta2*
Xentro_beta2*
Galgal_alpha2
Xentro_alpha2
Lepocu_alpha2
Chipun_alpha2
Parata_chr14
Petmar_chrm31
Petmar_chrm6
Parata_chr9
Parata_chr13
Petmar_chrm29
Parata_chr4
Petmar_chrm17
Parata_chr10
Parata_chr1
Petmar_chrm33
Petmar_chrm53
Xentro_alpha1
Galgal_alpha1
Lepocu_alpha1
Chipun_alpha1
Ptyfla_NA
Sackow_NA
Strpur_NA
Bralan_NA
2

CLGB_C20.contree

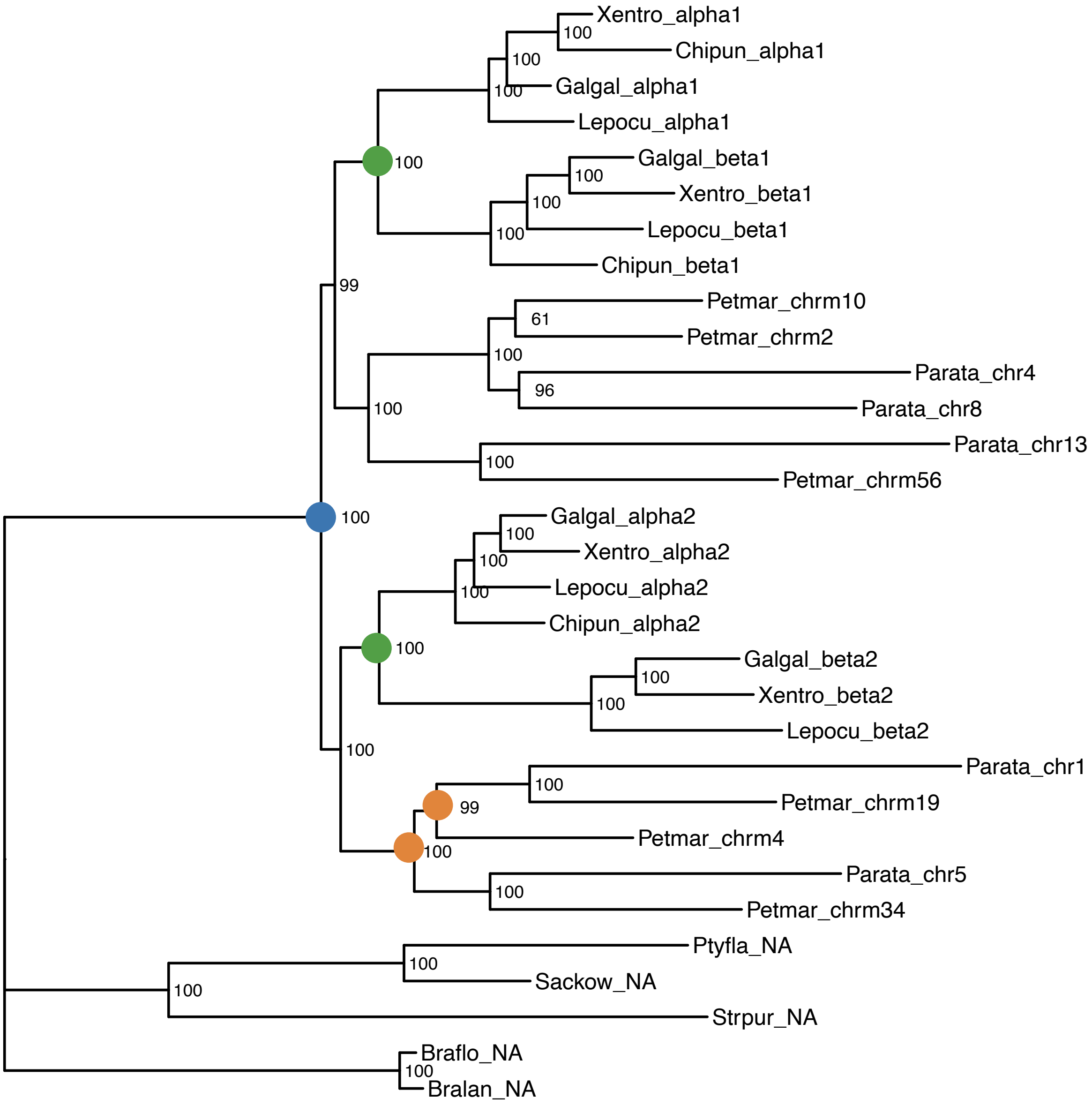

CLGC_C20.contree

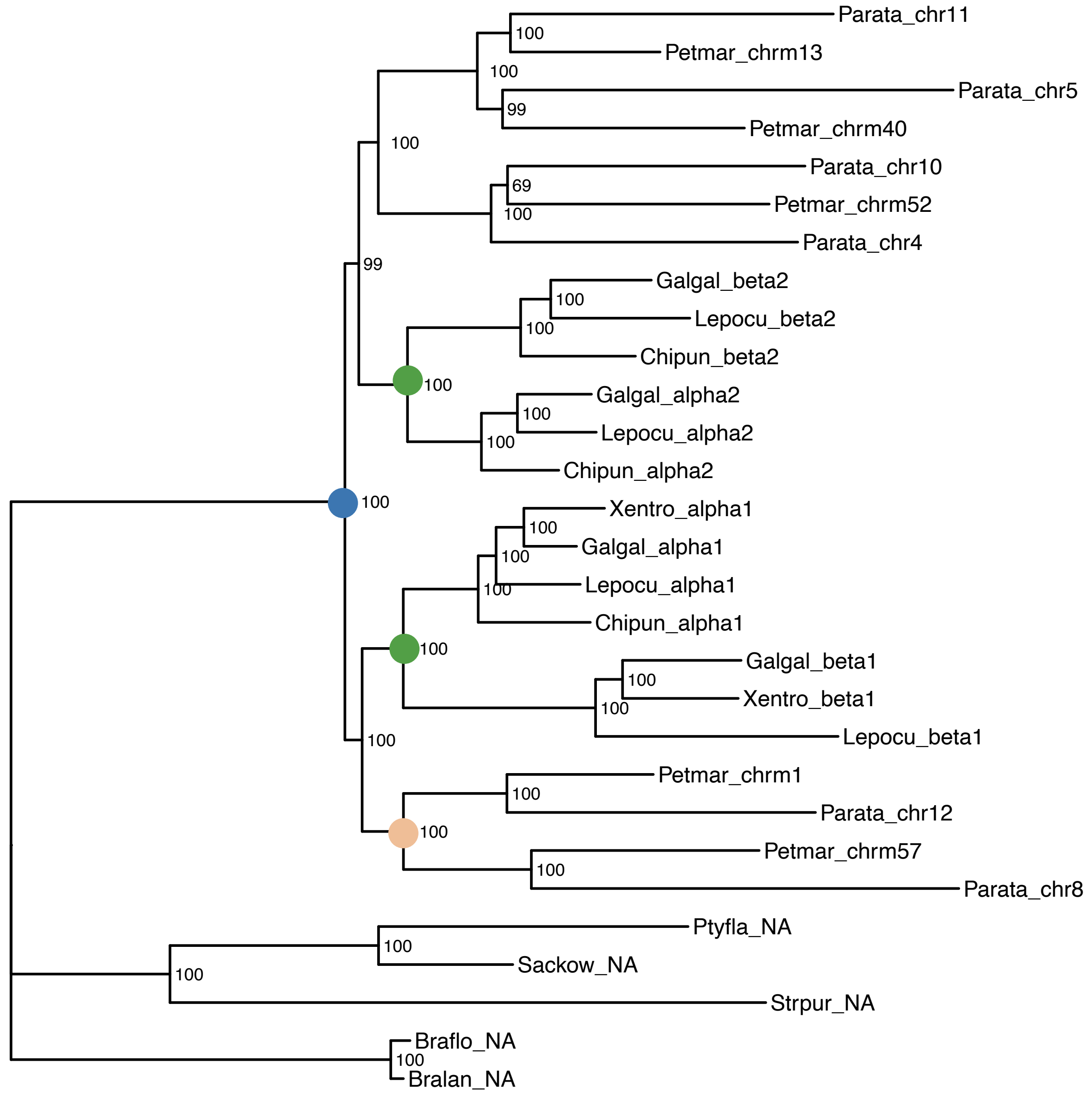

CLGD_C20.contree

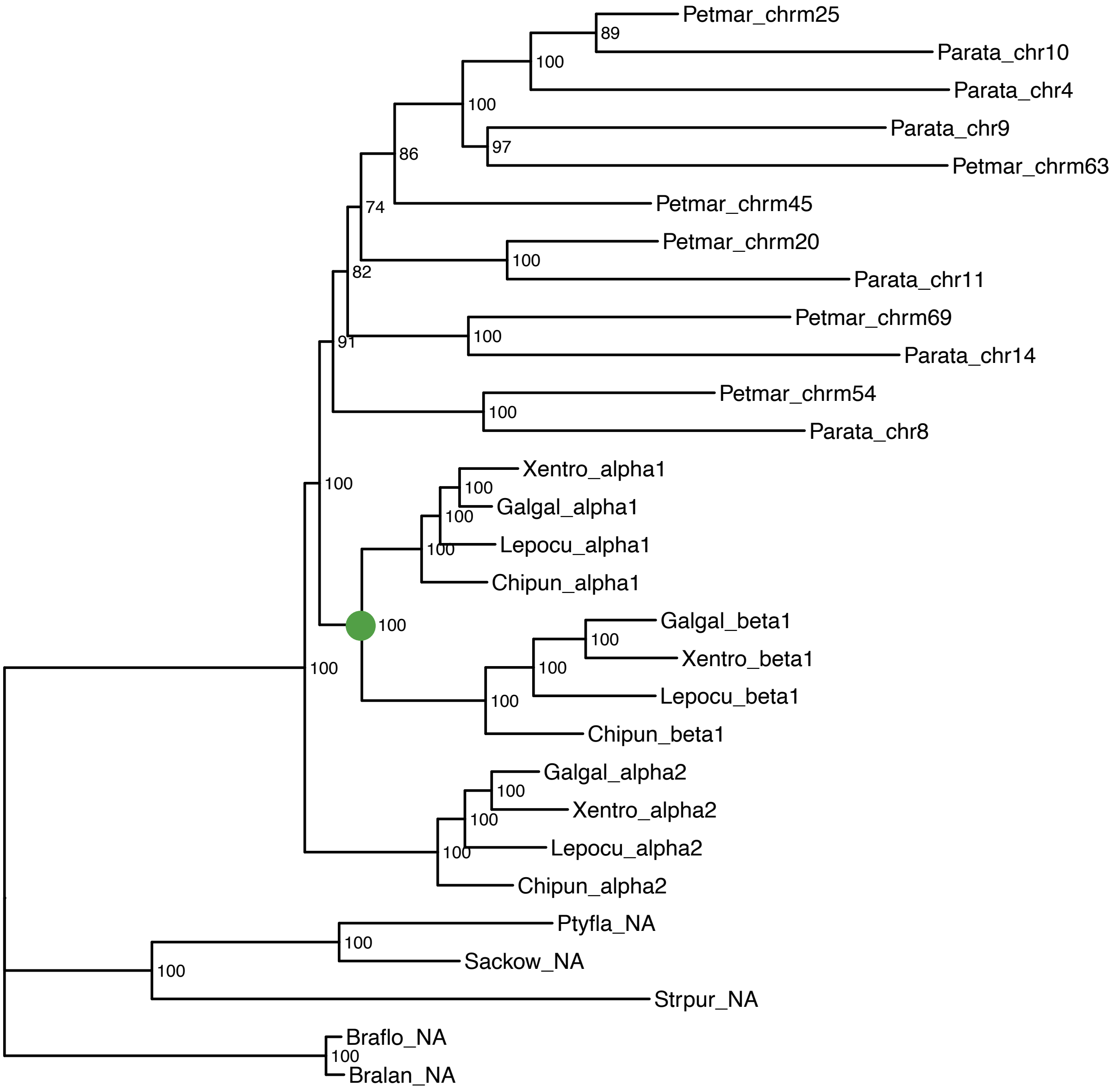

CLGE_C20.contree

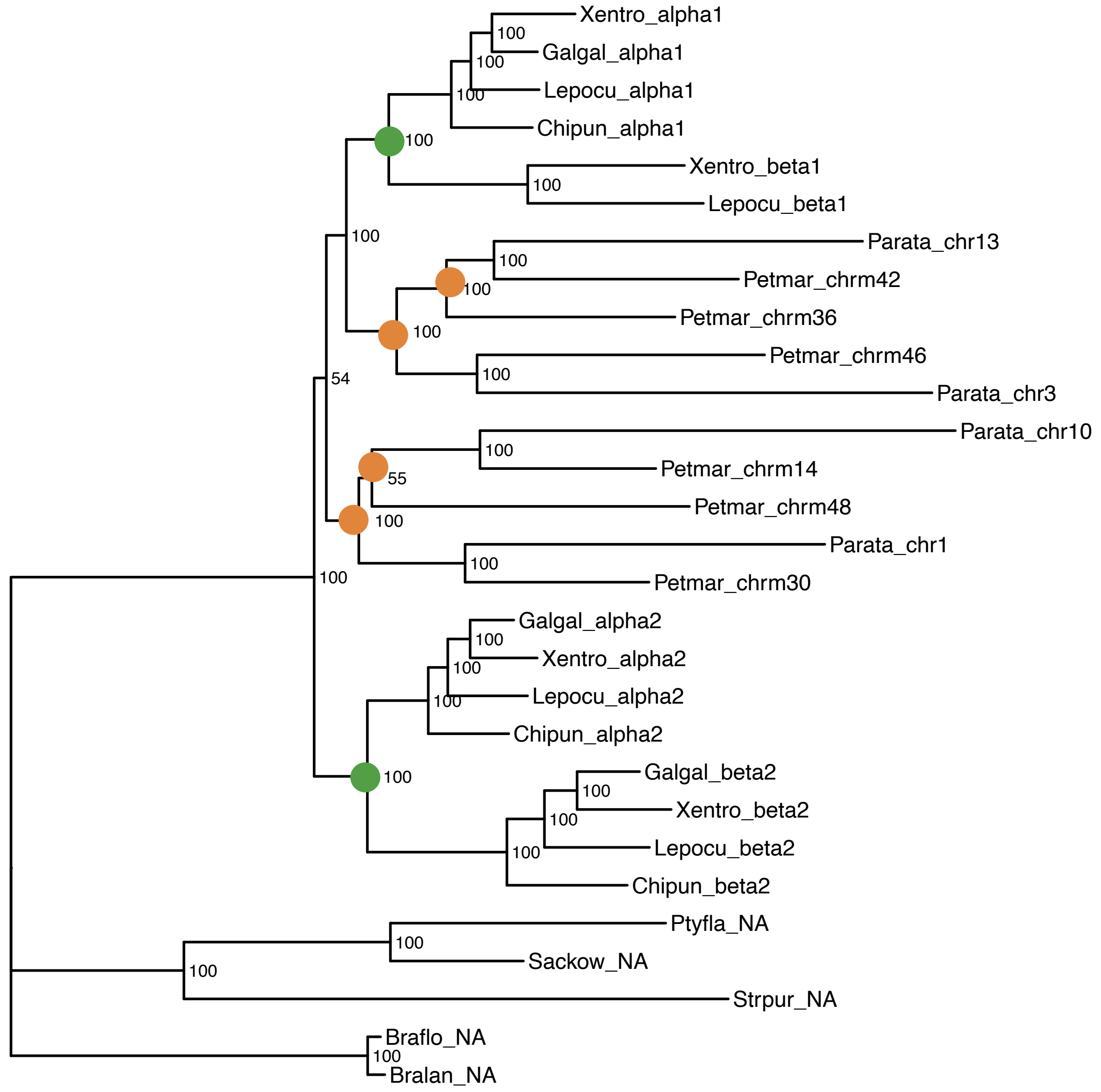

CLGF_C20.contree

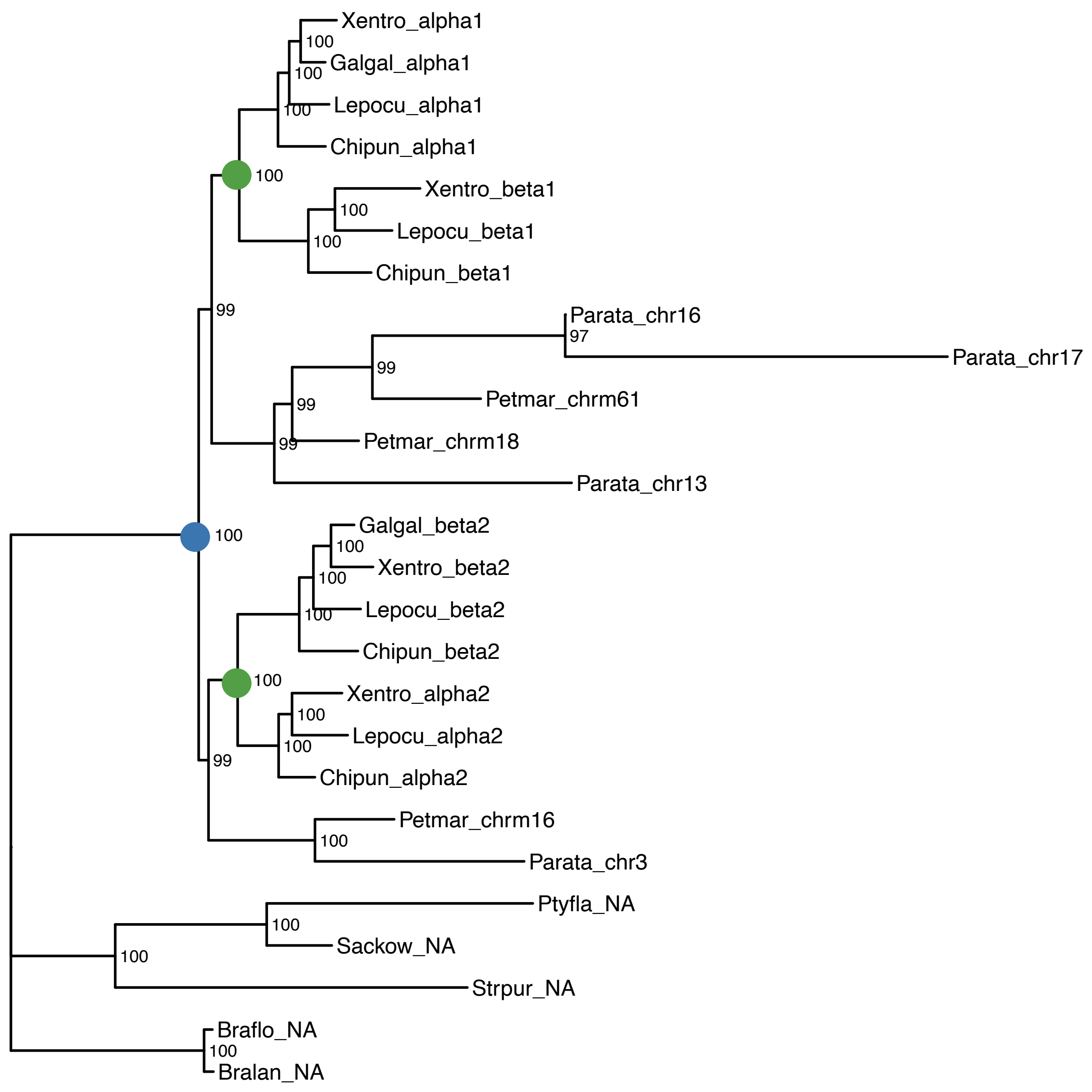

CLGG_C20.contree

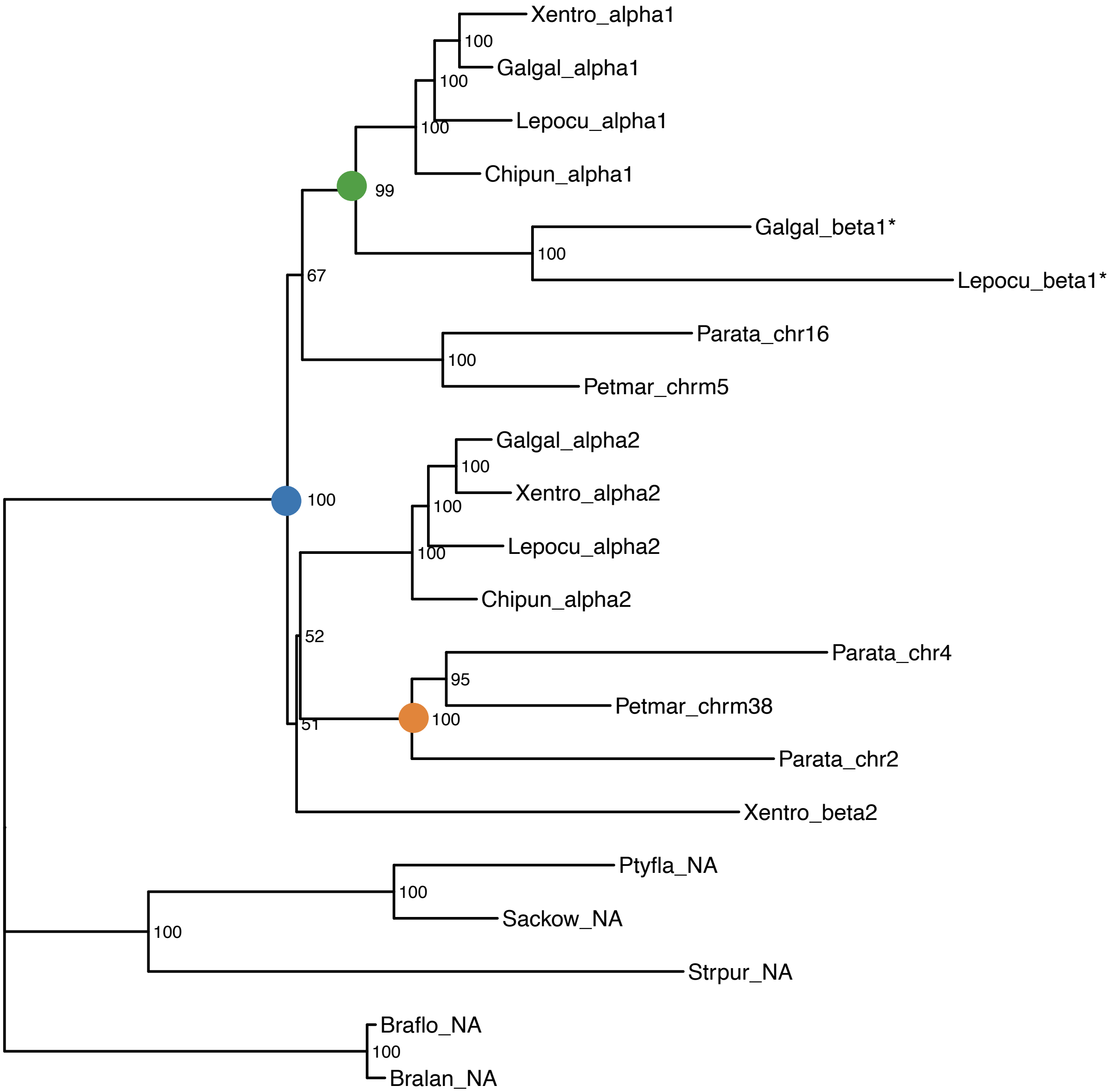

CLGH_C20.contree

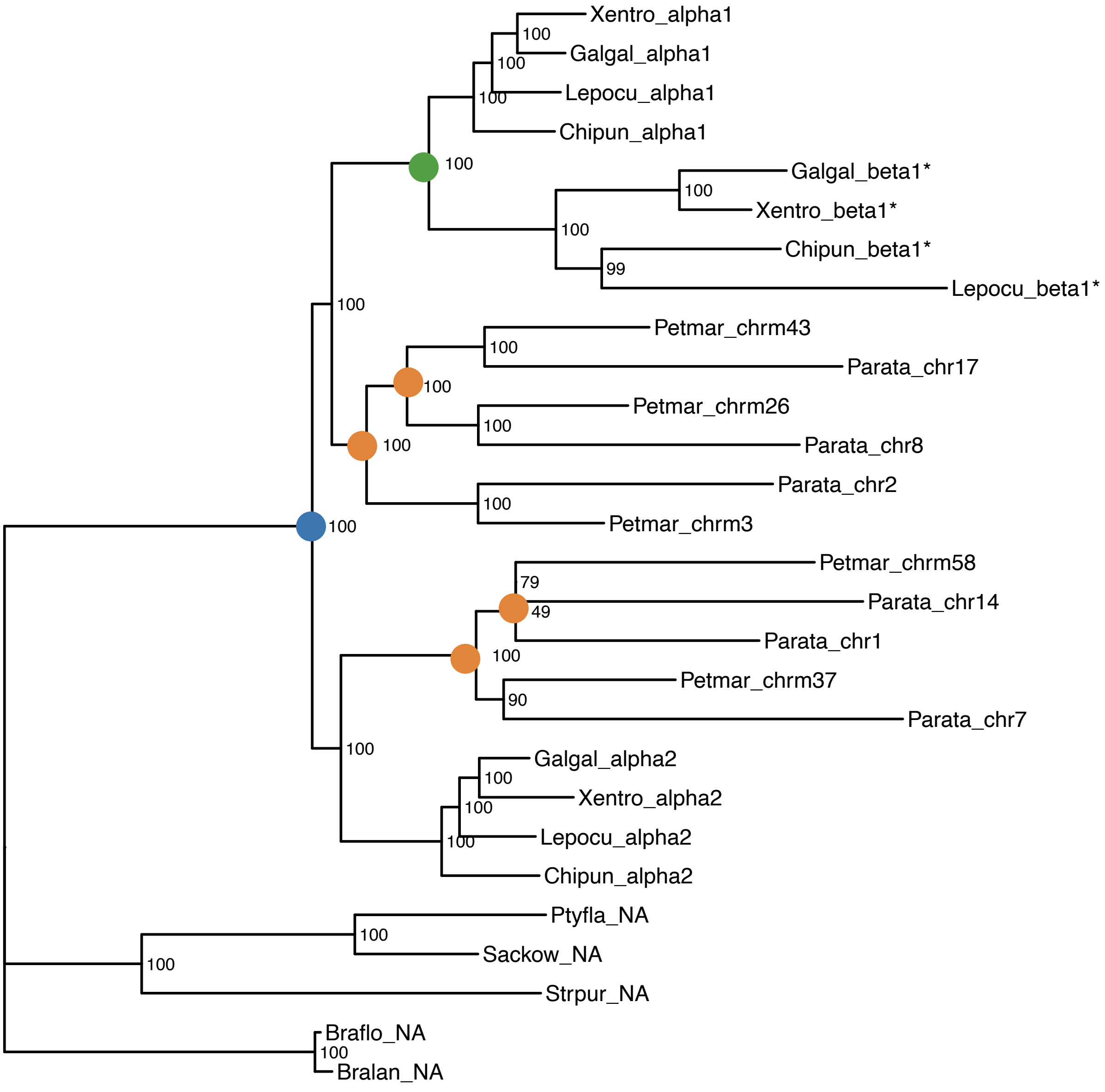

CLGI_C20.contree

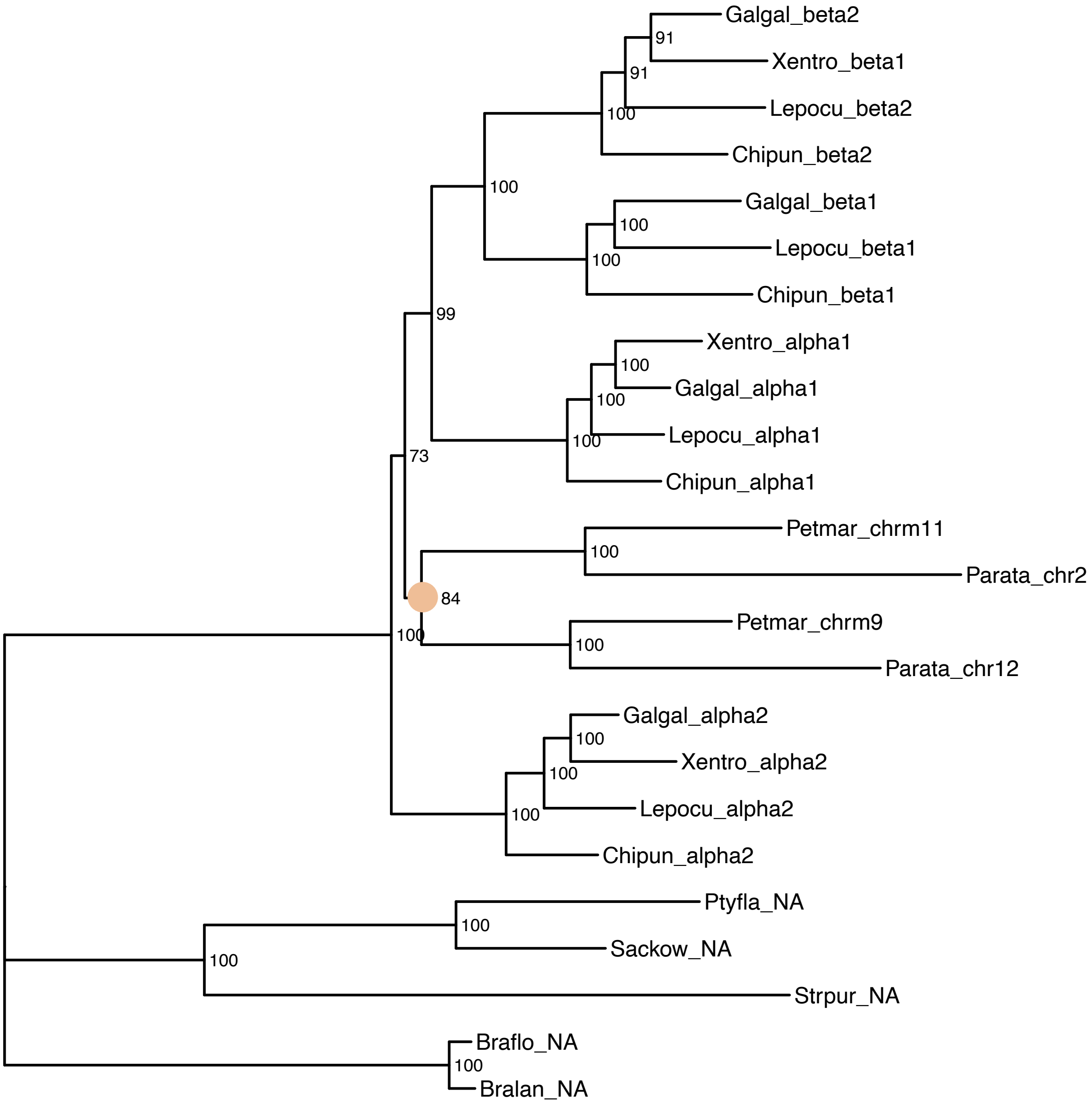

CLGJ_C20.contree

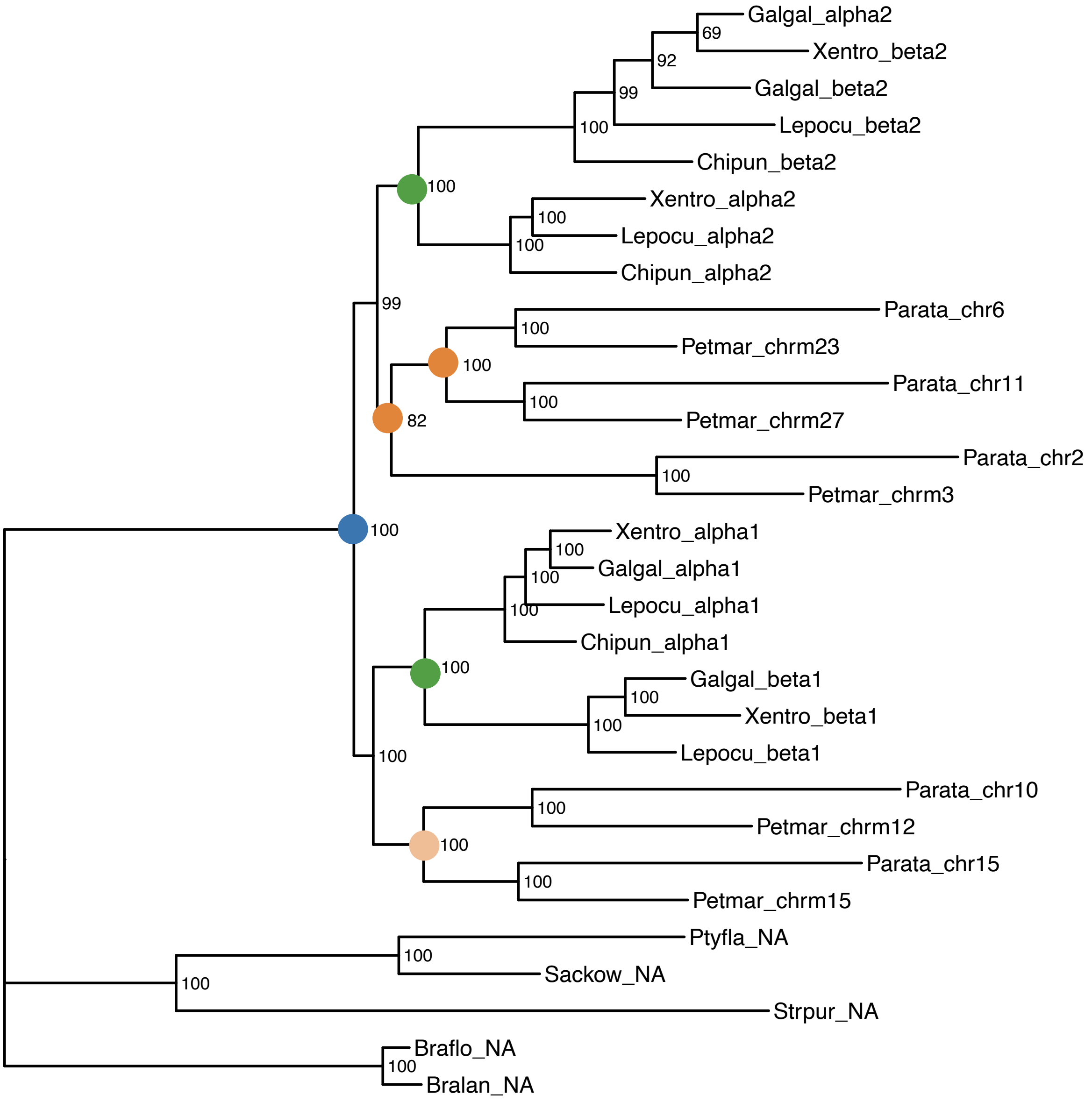

CLGK_C20.contree

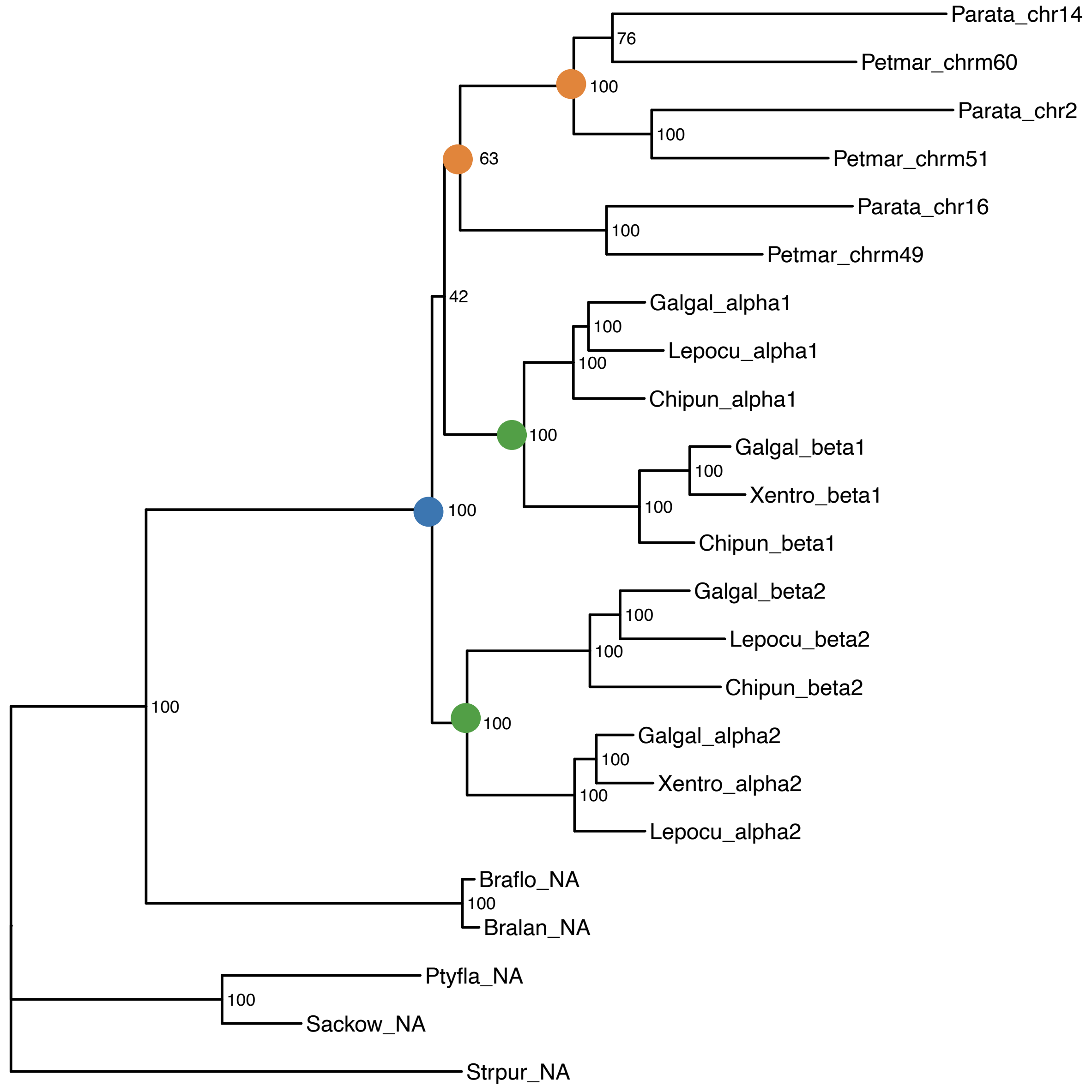

CLGL_C20.contree

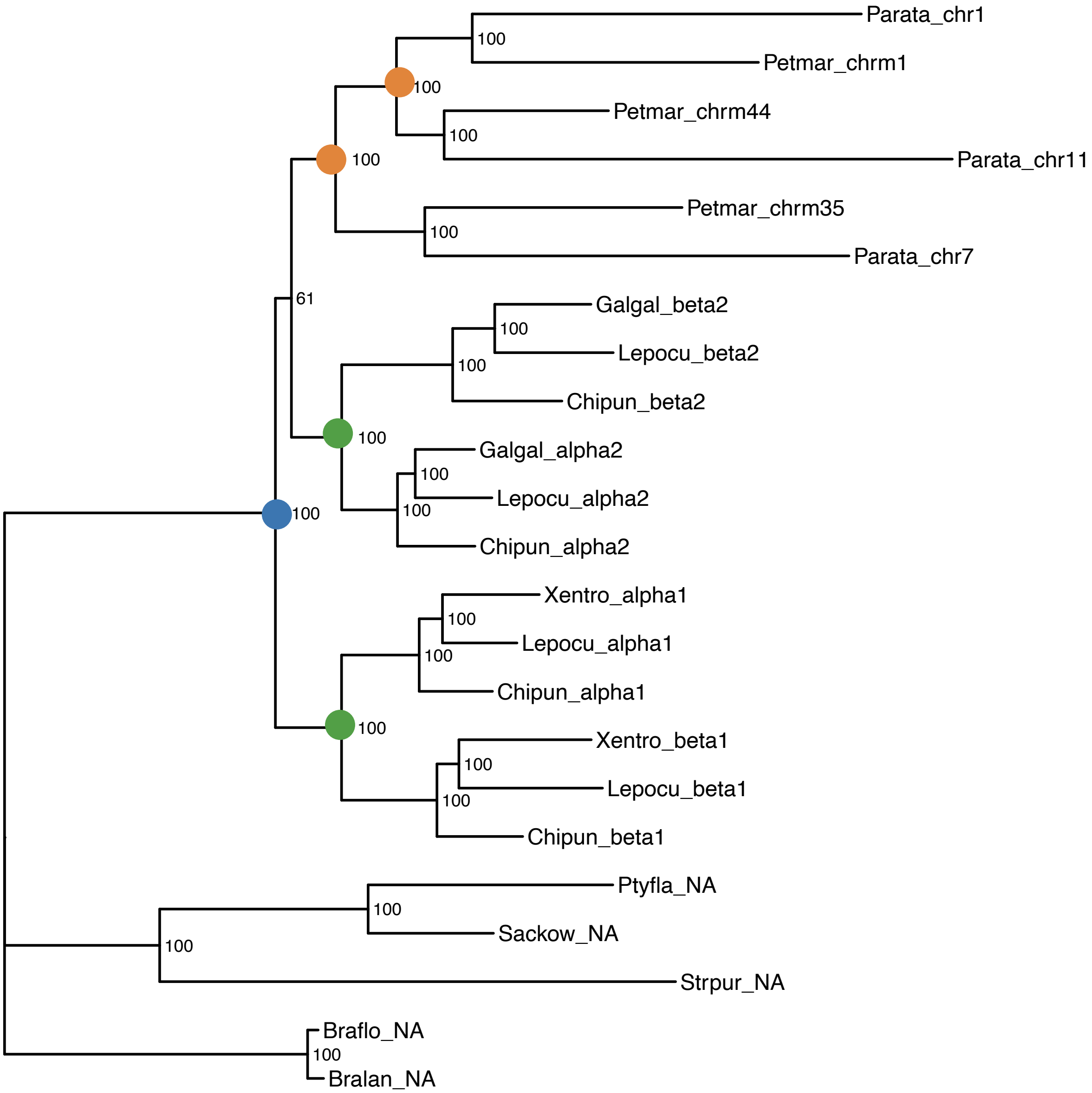

CLGM_C20.contree

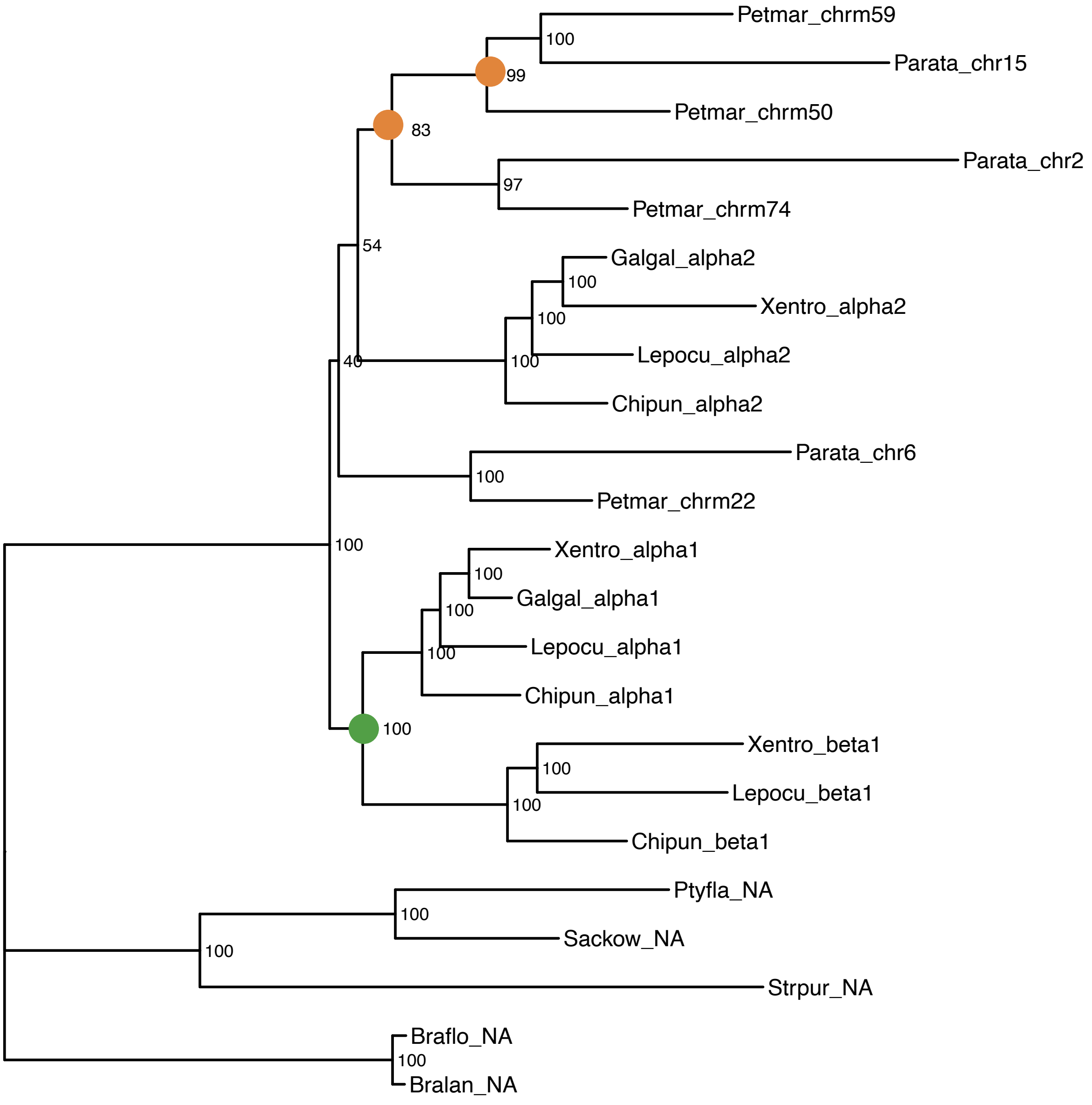

CLGN_C20.contree

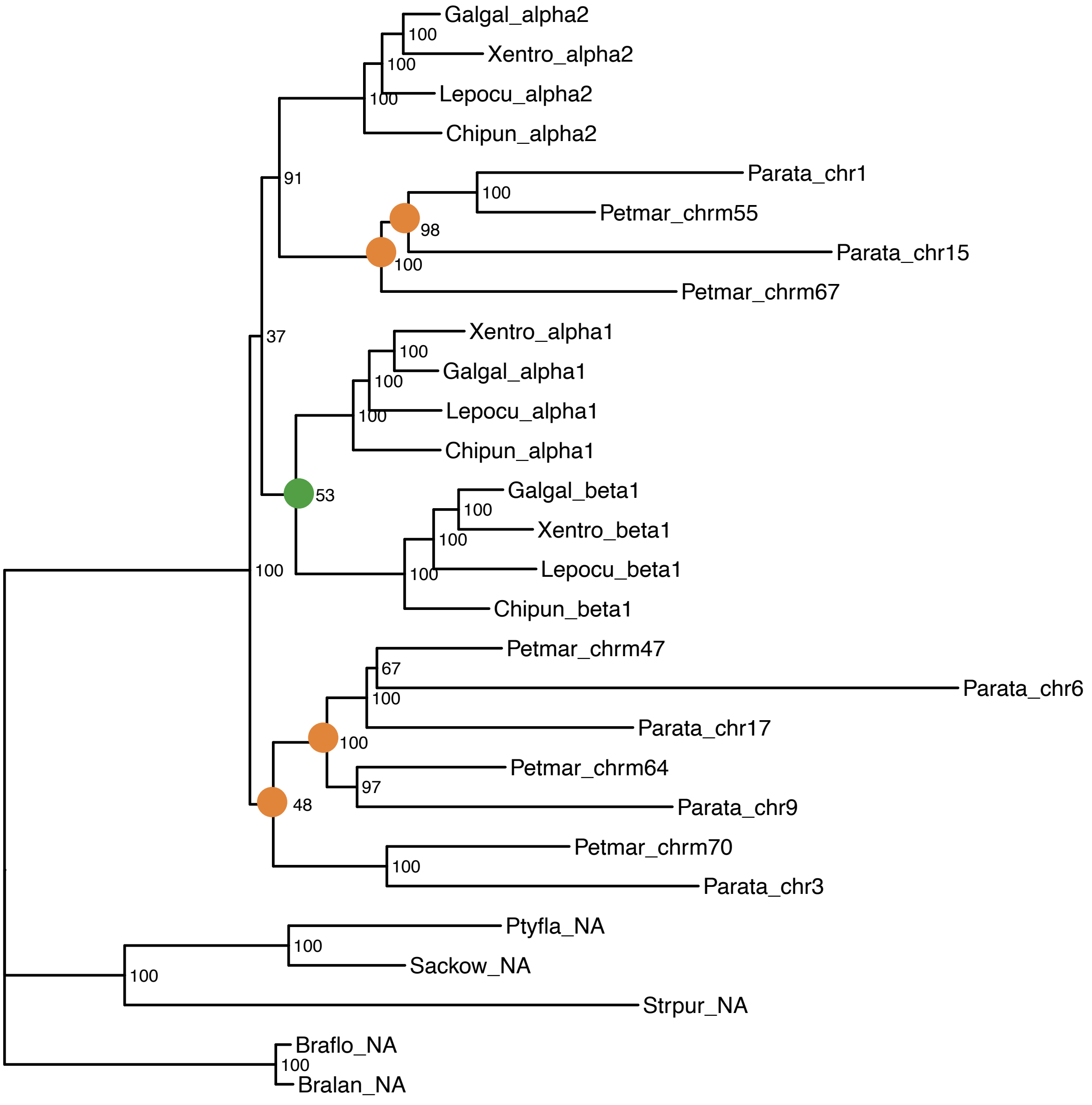

CLGO_C20.contree

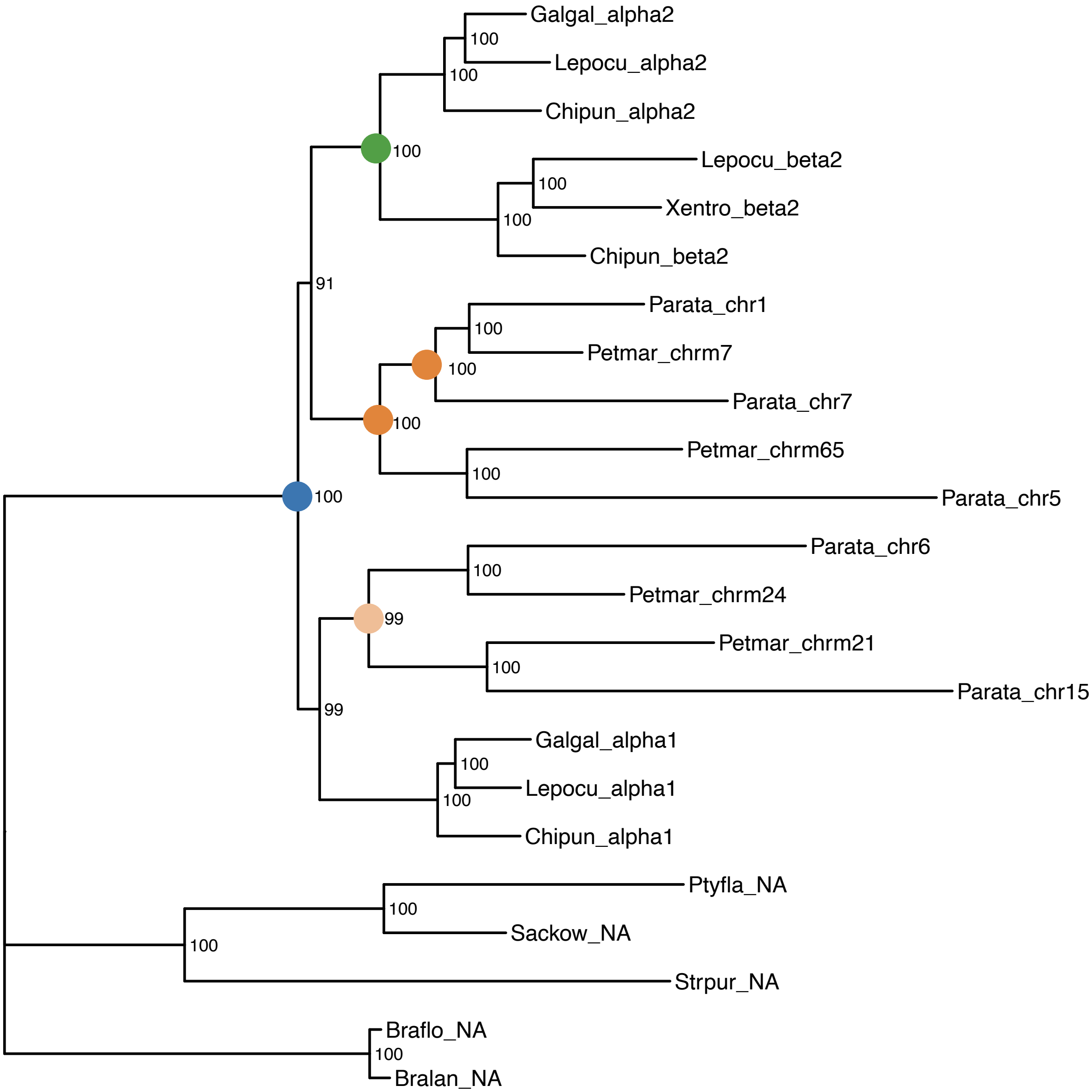

CLGP_C20.contree

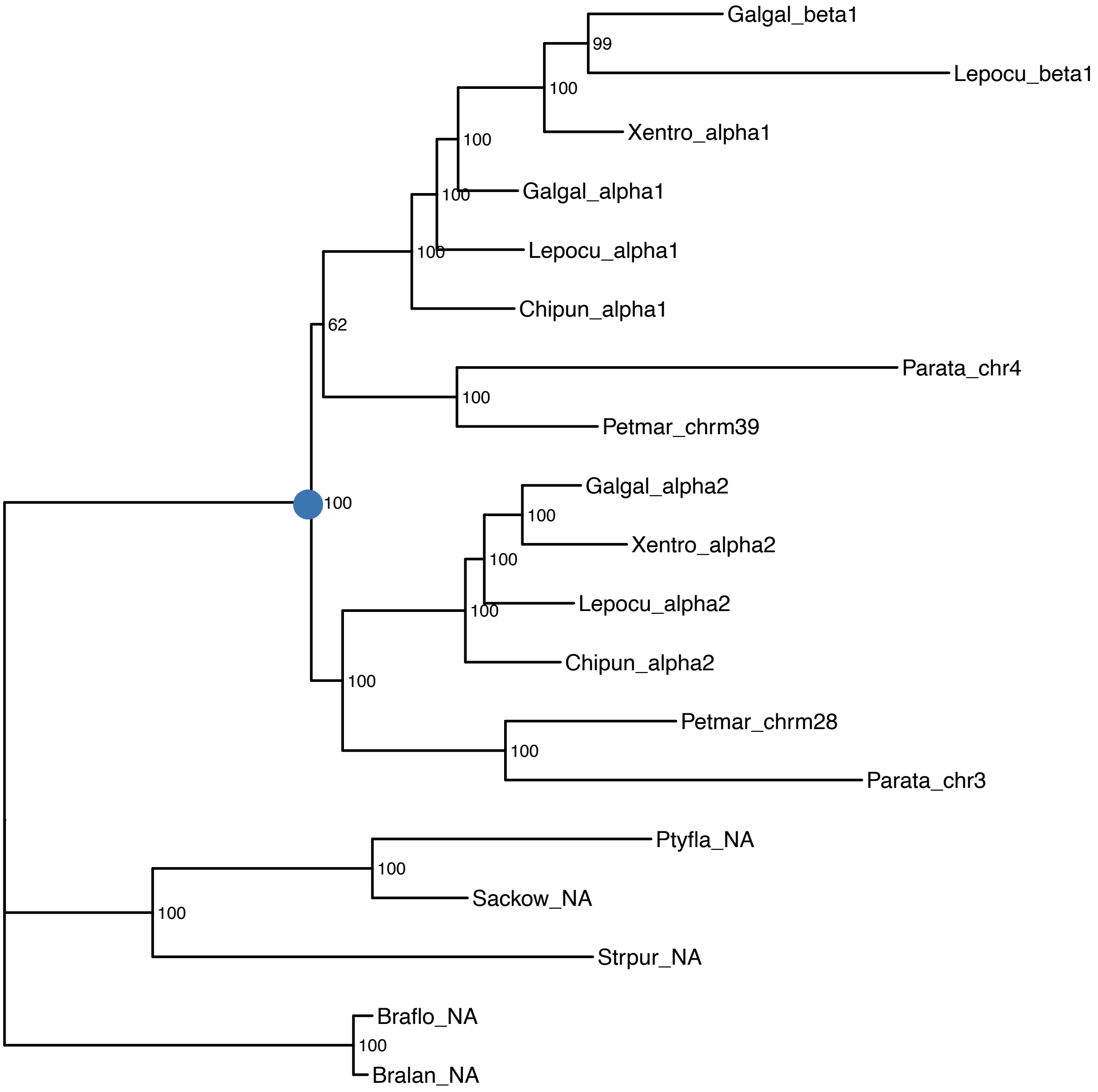

CLGQ_C20.contree

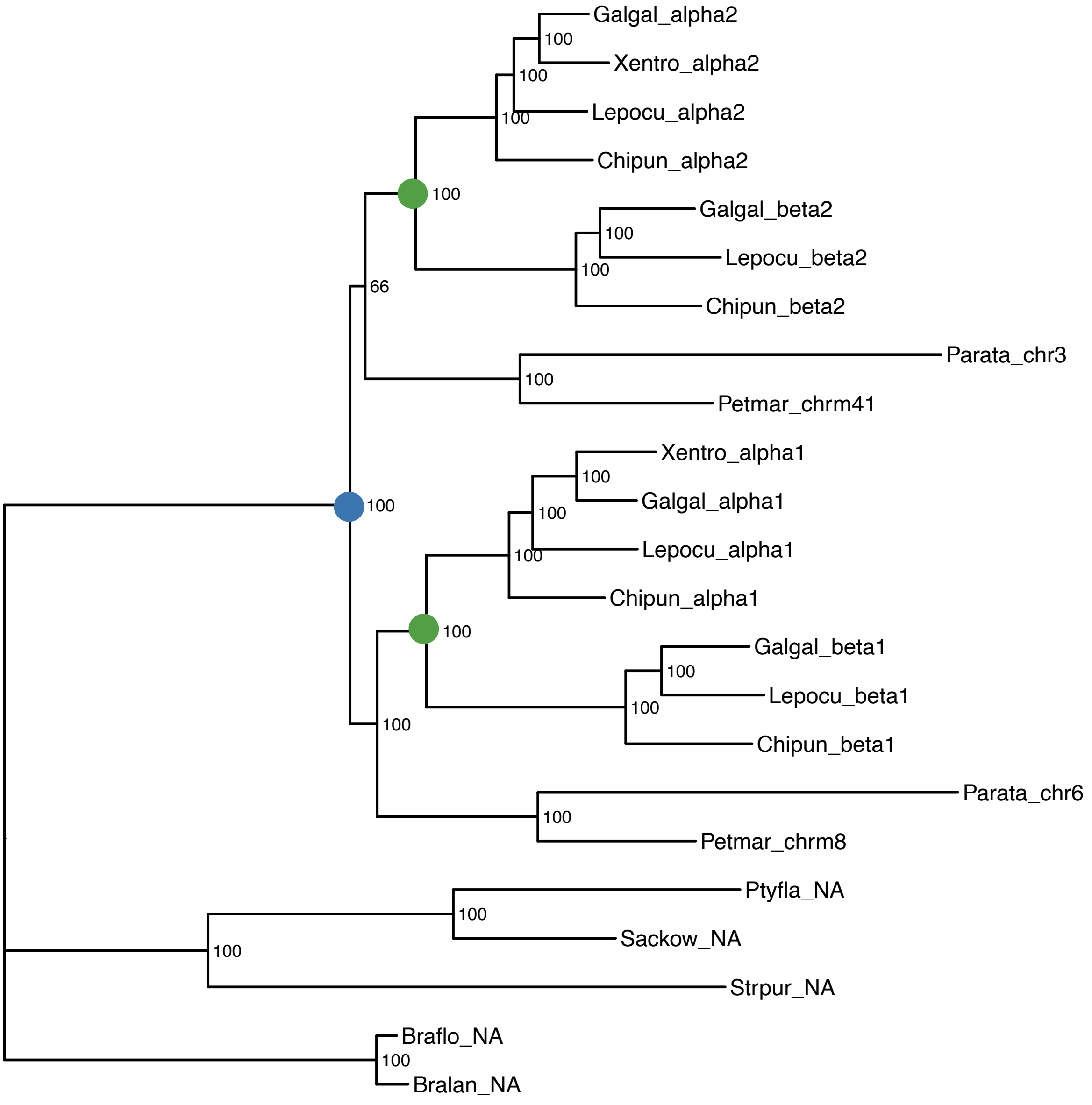

Supplement: Supplementary file 1 — This file contains Supplementary Notes 1–3, Supplementary References, and Supplementary Fig. 1 (Phylogenetic trees inferred for paralogons in each CLG assuming the C20+R model). [file 41586_2024_7070_MOESM1_ESM.pdf]
