# Supplementary material for: The hagfish genome and the evolution of vertebrates
Source: Nature. 2024 Jan 23;627(8005):811–20. doi: 10.1038/s41586-024-07070-3 (PMC10972751; doi:10.1038/s41586-024-07070-3)
Supplement: Supplementary file 2 — Reporting Summary [file 41586_2024_7070_MOESM2_ESM.pdf]

nature portfolio

Corresponding author(s): F. Marlétaz, D.S. Rokhsar, J.J. Smith

Last updated by author(s): Jan 4, 2024

Reporting Summary

Nature Portfolio wishes to improve the reproducibility of the work that we publish. This form provides structure for consistency and transparency in reporting. For further information on Nature Portfolio policies, see our Editorial Policies and the Editorial Policy Checklist.

Statistics

For all statistical analyses, confirm that the following items are present in the figure legend, table legend, main text, or Methods section.

n/a	Confirmed	
☒	☐	The exact sample size (*n*) for each experimental group/condition, given as a discrete number and unit of measurement
☒	☐	A statement on whether measurements were taken from distinct samples or whether the same sample was measured repeatedly
☐	☒	The statistical test(s) used AND whether they are one- or two-sided<br>*Only common tests should be described solely by name; describe more complex techniques in the Methods section.*
☒	☐	A description of all covariates tested
☐	☒	A description of any assumptions or corrections, such as tests of normality and adjustment for multiple comparisons
☐	☒	A full description of the statistical parameters including central tendency (e.g. means) or other basic estimates (e.g. regression coefficient) AND variation (e.g. standard deviation) or associated estimates of uncertainty (e.g. confidence intervals)
☐	☒	For null hypothesis testing, the test statistic (e.g. *F*, *t*, *r*) with confidence intervals, effect sizes, degrees of freedom and *P* value noted<br>*Give P values as exact values whenever suitable.*
☐	☒	For Bayesian analysis, information on the choice of priors and Markov chain Monte Carlo settings
☒	☐	For hierarchical and complex designs, identification of the appropriate level for tests and full reporting of outcomes
☒	☐	Estimates of effect sizes (e.g. Cohen's *d*, Pearson's *r*), indicating how they were calculated

Our web collection on statistics for biologists contains articles on many of the points above.

Software and code

Policy information about availability of computer code

Data collection	No software used for data collection
Data analysis	Assembly: Meraculous (v2.2.2.5) , PBJelly (v15.8.24), Meryl (v1.1), Genomescope2<br>Annotation: STAR (v2.5.2b), Stringtie (v1.3.3b), Trinity (v2.11.0), quiver (v2.0.0), GMAP (v. 2018-03-25), Mikado (v1.2.1), Portcullis (v1.0.2), Trans-decoder (v5.5.0), RepeatModeler (v1.0.11), RepeatMasker (v4.0.7), PASA pipeline (v2.3.1).<br>Phylogenetics: OMA tool (v2.4.1), Hmmer (v3.1b2), Msaprobs (0.9.7), HmmCleaner (v1b), BMGE (v1.12),IQ-TREE (v2.1.1), Phylobayes (v4.1e).<br>Gene family analyses: MMSeqs2 (r12-113e3), Broccoli (v1.1), MAFFT (v7.305), generax (v1.2.2), WHALE (v2.1.0), RAxML-NG v. 1.1 , miniprot 0.5-r179 ,<br>Transcriptomics: featureCount from subreads package (v1.6.3), WGCNA (v1.7.0)<br>DNA elimination: BWA-mem (v0.7.5a-r416), DifCover (Version 4), Jellyfish (v2.2.4), CD-HIT-EST (v4.6)<br><br>Custom code available at https://github.com/fmarletaz/hagfish

For manuscripts utilizing custom algorithms or software that are central to the research but not yet described in published literature, software must be made available to editors and reviewers. We strongly encourage code deposition in a community repository (e.g. GitHub). See the Nature Portfolio guidelines for submitting code & software for further information.

Data

Policy information about availability of data

All manuscripts must include a data availability statement. This statement should provide the following information, where applicable:

- Accession codes, unique identifiers, or web links for publicly available datasets
- A description of any restrictions on data availability
- For clinical datasets or third party data, please ensure that the statement adheres to our policy

Raw and processed sequences have been deposited in NCBI SRA (PRJNA953751) and Gene Expression Omnibus (GSE230176). The RNA-seq of Myxine glutinosa is available on the SRA (SRR25213276). The resequenced somatic tissues are also available on the SRA (Blood: SRR24133795 and Testes: SRR24130678). RNA-seq datasets used for comparative analyses are publicly available for Japanese lamprey (PRJNA354821, PRJNA349779, PRJNA312435), gar (PRJNA255881) and amphioxus (PRJNA416977) as well as the sea lamprey (PRJNA497902).

Field-specific reporting

Please select the one below that is the best fit for your research. If you are not sure, read the appropriate sections before making your selection.

☒ Life sciences ☐ Behavioural & social sciences ☐ Ecological, evolutionary & environmental sciences

For a reference copy of the document with all sections, see nature.com/documents/nr-reporting-summary-flat.pdf

Life sciences study design

All studies must disclose on these points even when the disclosure is negative.

Sample size	No statistical method was used for determining sample size.
Data exclusions	No data exclusions.
Replication	No such experiments.
Randomization	No randomization was used
Blinding	Blinding was not relevant to our study as comparison were performed by computer software not influenced by investigator.

Reporting for specific materials, systems and methods

We require information from authors about some types of materials, experimental systems and methods used in many studies. Here, indicate whether each material, system or method listed is relevant to your study. If you are not sure if a list item applies to your research, read the appropriate section before selecting a response.

Materials & experimental systems

n/a	Involved in the study	
☒	☐	Antibodies
☒	☐	Eukaryotic cell lines
☒	☐	Palaeontology and archaeology
☐	☒	Animals and other organisms
☒	☐	Human research participants
☒	☐	Clinical data
☒	☐	Dual use research of concern

Methods

n/a	Involved in the study	
☒	☐	ChIP-seq
☒	☐	Flow cytometry
☒	☐	MRI-based neuroimaging

Animals and other organisms

Policy information about studies involving animals; ARRIVE guidelines recommended for reporting animal research

Laboratory animals	*For laboratory animals, report species, strain, sex and age OR state that the study did not involve laboratory animals.*
Wild animals	Animals were samples in Suruga Bay, off Yaizu (300-330m depth) and maintained in seawater aquariums at 11-13°C.
Field-collected samples	*For laboratory work with field-collected samples, describe all relevant parameters such as housing, maintenance, temperature, photoperiod and end-of-experiment protocol OR state that the study did not involve samples collected from the field.*

Ethics oversight	In agreement with procedures authorized by Guidelines for Proper Conduct of Animal Experiments by the Science Council of Japan (2006), animals were anaesthetized using Tricaine (MS222, Sigma) before sacrifice and dissection.

Note that full information on the approval of the study protocol must also be provided in the manuscript.
